# Impact of fatty acid binding protein 5-deficiency on COPD exacerbations and cigarette smoke-induced inflammatory response to bacterial infection

**DOI:** 10.1186/s40169-019-0227-8

**Published:** 2019-03-15

**Authors:** Deviyani M. Rao, Della T. Phan, Michelle J. Choo, Michael R. Weaver, Rebecca E. Oberley-Deegan, Russell P. Bowler, Fabienne Gally

**Affiliations:** 1 Department of Biomedical Research, National Jewish Health, 1400 Jackson St., Room K827, Denver, CO 80206 USA; 2 Department of Medicine, National Jewish Health, 1400 Jackson Street, Denver, CO 80206 USA; 3 Department of Biochemistry and Molecular Biology, University of Nebraska Medical Center, BCC 6.12.392, 985870 Nebraska Medical Center, Omaha, NE 68198-5870 USA

**Keywords:** FABP5, COPD exacerbations, Cigarette smoke, Inflammation

## Abstract

**Background:**

Although cigarette smoking (CS) is by far the most important risk factor of chronic obstructive pulmonary disease (COPD), repeated and sustained infections are clearly linked to disease pathogenesis and are responsible for acute inflammatory flares (i.e. COPD exacerbations). We have previously identified Fatty Acid Binding Protein 5 (FABP5) as an important anti-inflammatory protein in primary airway epithelial cells.

**Results:**

In this study we found decreased *FABP5* mRNA and protein levels in peripheral blood mononuclear cells (PBMCs) of COPD patients, especially among those who reported episodes of COPD exacerbations. Using wildtype (WT) and FABP5^−/−^ mice, we examined the effects of FABP5 on CS and infection-induced inflammatory responses. Similarly to what we saw in airway epithelial cells, infection increased FABP5 expression while CS decreased FABP5 expression in mouse lung tissues. CS-exposed and *P. aeruginosa*-infected FABP5^−/−^ mice had significantly increased inflammation as shown by increased lung histopathological score, cell infiltration and inflammatory cytokine levels. Restoration of FABP5 in alveolar macrophages using a lentiviral approach attenuated the CS- and bacteria-induced pulmonary inflammation. And finally, while *P. aeruginosa* infection increased PPARγ activity, CS or FABP5 knockdown greatly reduced PPARγ activity.

**Conclusions:**

These findings support a model in which CS-induced FABP5 inhibition contributes to increased inflammation in COPD exacerbations. It is interesting to speculate that the increased inflammation is a result of decreased PPARγ activity.

## Background

Chronic obstructive pulmonary disease (COPD) is characterized by chronic airway inflammation, irreversible airflow limitation and emphysema [1]. The main risk factor of COPD is cigarette smoke (CS) [2]. COPD is presently the fourth leading cause of death worldwide, but it is predicted to become the third by 2030 according to the world health organization [3]. Bacterial and viral infections have been implicated as a major cause of COPD exacerbations. *Pseudomonas aeruginosa* (*P. aeruginosa*) is often disregarded as a COPD pathogen. However, it represents 5–10% of the pathogens that colonize COPD lungs [4, 5] and is not only involved in acute exacerbations, but also contributes to the chronic process of the disease [6]. In addition, several studies have shown that *P. aeruginosa* is the cause of more infections as severity of COPD increases [7–9]. In hospitals, the organism contaminates moist/wet reservoirs such as respiratory equipment and indwelling catheters. Infections can occur in almost every body site but are particularly serious in the bloodstream.

Since the majority of COPD patients are current or former smokers, it is thought that CS has immunosuppressive effects that increase patients’ susceptibility to infections. To discover novel innate immune genes regulated by CS in humans, we took advantage of the non-biased *C. elegans* model. Using microarray and RNAi, we successfully identified *lbp*-*7*, a lipid binding protein, which was down-regulated after CS exposure and played a role in innate immunity [10]. Interestingly the human orthologue of this protein, FABP5, was up-regulated in response to bacterial infection, and was down-regulated in COPD patients as compared to healthy smokers [10]. We further showed that FABP5 exerts immunomodulatory functions in the airway epithelium against CS exposure and subsequent bacterial infection through its modulation of the nuclear receptor peroxisome proliferator-activated receptor (PPAR)-γ activity [11]. However, the anti-inflammatory function of FABP5 in COPD exacerbations is still unknown.

In this study we examined the effects of CS on inflammatory responses in vivo to determine the role of FABP5 in these processes. We also looked at the role of FABP5 in macrophages since these cells are at the center of the processes required for the resolution of inflammation, including engulfment of apoptotic cells, activation of PPARγ, and repair processes [12, 13]. We hypothesized that cigarette smoke decreases FABP5 expression, thus impairing its anti-inflammatory function and contributing to a more sustained bacterial-induced inflammation. To test this hypothesis, we measured FABP5 expression and PPARγ activation in peripheral blood mononuclear cells (PBMCs) of patients with or without COPD and characterized the relation between FABP5 suppression and disease exacerbations. In addition, we evaluated the effects of CS on FABP5 expression and PPARγ activity and defined the role of FABP5 in a murine model of COPD exacerbation and in macrophages.

## Results

**Fig. 1:**
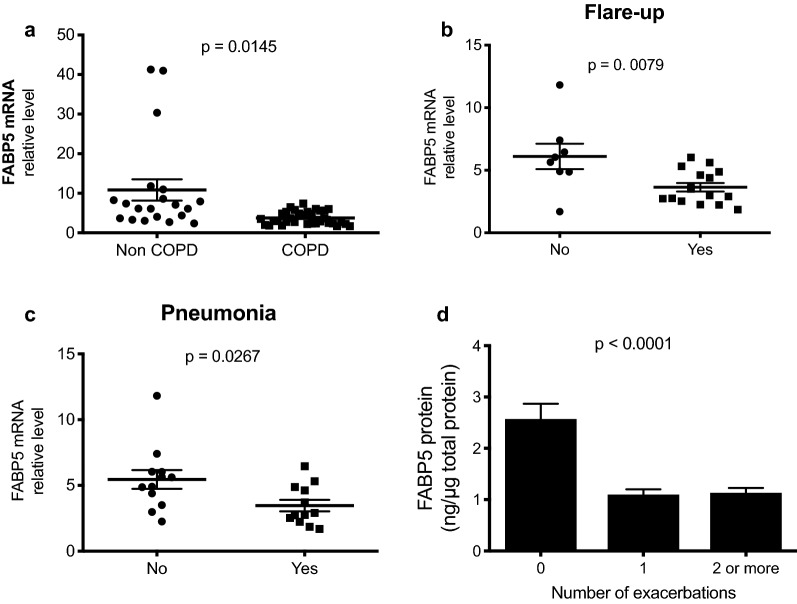
FABP5 expression is decreased in PBMCs of COPD patients and further decreased in COPD exacerbations. **a** Real-time PCR for human *FABP5* was performed on 20 non-COPD donors and 34 COPD donors (GOLD 1–4). Association of *FABP5* mRNA levels in 34 COPD donors who reported. **b** Flare-up and **c** pneumonia. **d** FABP5 protein expression as a function of exacerbation numbers among COPD patients. Data in **a**–**c** represent the individual patient’s distribution along with the mean ± SEM. Data in **d** represent the mean ± SEM of FABP5 protein concentration in function of the self-reported number of exacerbations in the past 12 months

We extracted the mRNA from freshly isolated PBMCs of 20 non-COPD patients and 36 COPD patients enrolled in the NIH-sponsored COPDGene study cohort [14]. Levels of *FABP5* mRNA were significantly reduced in patients with COPD compared to non-COPD as measured by real-time RT-PCR (Fig. 1a). Furthermore, *FABP5* mRNA expression was further decreased in COPD patients who reported flare-up and pneumonia episodes (Fig. 1b, c) within the last 12 months. We also showed a significant decrease in FABP5 protein levels among COPD patients who reported one or more exacerbation as compared to patients who did not report any exacerbation in the last 12 months (Fig. 1d). Taken together, these data indicate that FABP5 expression is decreased in patients with COPD and further decreased in patients reporting episodes of COPD exacerbation.

**Fig. 2:**
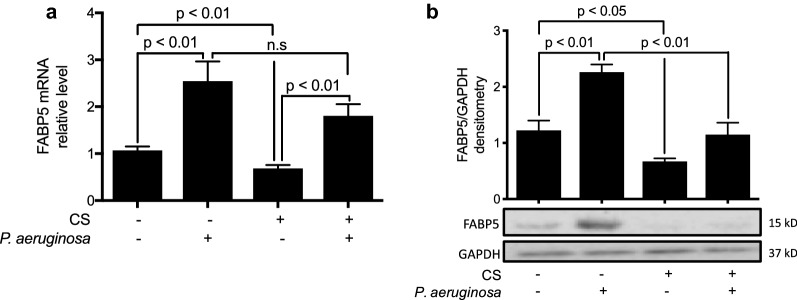
Cigarette smoke decreases infection-induced FABP5 expression in mouse lung tissues. **a** *Fabp5* mRNA expression following 5 days of CS exposure and *P. aeruginosa* infection in the lung tissues of WT mice. **b** Western blot evaluation and densitometry quantification of FABP5 protein in the lung tissues of WT mice. n = 5 mice per group. Data are representative of 3 independent experiments and represent the mean ± SEM

To define the role of FABP5 in CS- and exacerbation-induced responses, we measured the effects of CS and bacterial infection on FABP5 expression in wild type (WT) mice exposed to 5 days of CS or filtered air. As indicated in Fig. 2a, while *P. aeruginosa* infection increased *FABP5* mRNA expression, CS exposure alone decreased *FABP5* expression in the lung homogenates of WT mice. The combination of both CS exposure and *P. aeruginosa* infection resulted in similar *FABP5* mRNA levels as observed for the infection alone in the lung homogenates of WT mice (Fig. 2a). Western blot analysis of FABP5 protein in lung homogenates further confirmed that FABP5 expression is increased after *P. aeruginosa* infection and suppressed after CS exposure (Fig. 2b). These results confirm that FABP5 expression is positively regulated during bacterial infection, but CS exposure inhibits FABP5 expression in murine lung tissues.

**Fig. 3:**
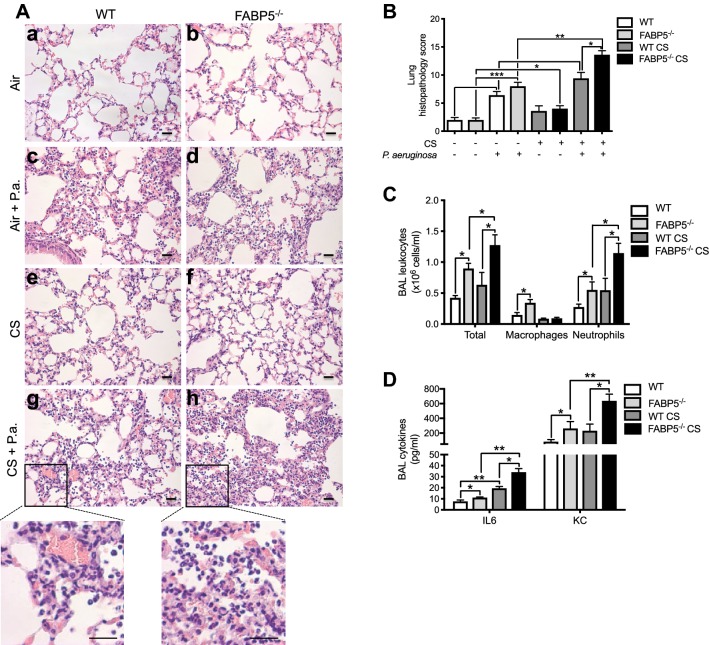
FABP5-deficiency increases CS- and *P. aeruginosa*-induced pulmonary inflammation. After 5 days of CS exposure, mice were infected with *P. aeruginosa* and harvested 16 h post infection. **A** Hematoxylin and eosin stained paraffin sections of lungs from WT and FABP5^−/−^ mice. Bars represent 50 µm. Insert: Higher magnification. **B** Lung tissue histopathology score of WT or FABP5^−/−^ mice. **C** Total leukocytes, macrophages and neutrophils in the bronchoalveolar lavage (BAL) of WT and FABP5^−/−^ mice. **D** IL-6 and KC protein levels in the BAL of WT and FABP5^−/−^ mice exposed to filtered air or CS 16 h post infection. Data are representative of three independent experiments and represent the mean ± SEM. n = 4–6 mice/group. *p < 0.05, **p < 0.01, ***p < 0.001

To assess the impact of FABP5-deficiency on CS exposure and bacterial-induced inflammation, WT and FABP5^−/−^ mice were exposed to CS 5 h a day for 5 days. Control mice were exposed to room air. Mice were then intranasally inoculated with 10^7^ CFU of viable *P. aeruginosa*. To address whether FABP5^−/−^ mice displayed increased inflammation, we performed histologic assessment of lung tissues. No overt inflammation was observed in air-exposed saline-treated WT and FABP5^−/−^ mice (Fig. 3A a, b). Sixteen hours post inoculation with *P. aeruginosa*, we observed similar perivascular and peribronchial inflammatory infiltrates in both WT and FABP5^−/−^ mice (Fig. 3A c, d) as illustrated by increased lung histopathology score compared to air-exposed saline-treated mice (Fig. 3B). CS exposure of saline-treated WT and FABP5^−/−^ mice showed minimal increase of inflammatory infiltrates, mainly mononuclear cells (Figs. 3A e, f). The combination of CS exposure and bacterial infection led to increased inflammation compared to infection alone (Figs. 3A g, h), with a significant worsening of lung histopathology score in FABP5^−/−^ mice compared to WT mice (Fig. 3B).

We next examined the cellular content (Fig. 3C) and inflammatory cytokine profiles (Fig. 3D) in the bronchoalveolar lavage (BAL) of *P. aeruginosa*-infected mice. As expected from the histologic assessment of lung tissues, we did not see a difference between WT or FABP5^−/−^ saline-treated air-exposed and saline-treated CS-exposed animals (data not shown). However, there was a significant increase in the numbers of neutrophils following *P. aeruginosa* in FABP5^−/−^ mice whether or not the mice were exposed to CS as compared to their WT counterpart (Fig. 3C). In addition, both inflammatory cytokines IL-6 and KC were increased in FABP5^−/−^ mice as compared to WT animals in all the different treatment groups (Fig. 3D). Taken together these data confirm that FABP5^−/−^ mice have increased inflammation following CS exposure and bacterial infection.

**Fig. 4:**
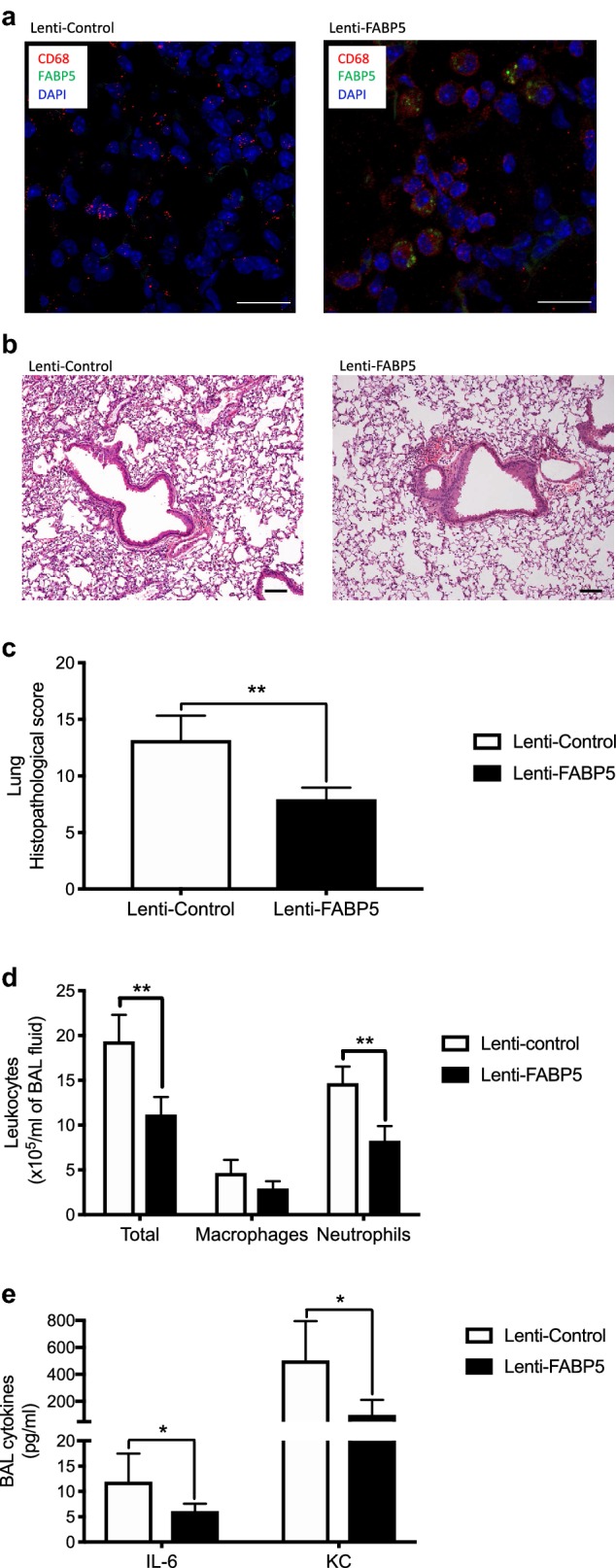
Restoration of FABP5 in alveolar macrophages is sufficient to reduce inflammation following CS and infection. FABP5^−/−^ mice were inoculated with FABP5 lentiviral vector (Lenti-FABP5) or empty vector (Lenti-Control). Two weeks later, mice were exposed to 5 days of CS and subsequently infected with *P. aeruginosa*. Mice were harvested 16 h post infection. **a** Immunostaining for FABP5 (green) and CD68 (red) in the lung tissue of FABP5^−/−^ mice inoculated with Lenti-Control or Lenti-FABP5. Bars represent 20 µm. **b** Total leukocytes, macrophages and neutrophils in the bronchoalveolar lavage (BAL) of FABP5^−/−^ mice inoculated with Lenti-Control or Lenti-FABP5. Bars represent 50 µm. **c** IL-6 and KC protein levels in the BAL of FABP5^−/−^ mice inoculated with Lenti-Control or Lenti-FABP5. **d** Hematoxylin and eosin stained paraffin sections of lungs from FABP5^−/−^ mice inoculated with Lenti-Control or Lenti-FABP5. Bars represent 100 µm. **e** Lung tissue histopathology score of FABP5^−/−^ mice inoculated with Lenti-Control or Lenti-FABP5. Data are representative of three independent experiments and represent the mean ± SEM. n = 5–6 mice/group. *p < 0.05, **p < 0.01

To determine whether FABP5 restoration in FABP5^−/−^ mice could protect them from CS and infection-induced inflammation, we performed in vivo alveolar macrophage FABP5 gene transfer in FABP5^−/−^ mice via intranasal inoculation of a lentiviral vector expressing mouse FABP5 (Lenti-FABP5) or the empty vector (Lenti-Control) (Fig. 4a). FABP5^−/−^ mice given Lenti-Control or Lenti-FABP5 were then exposed to CS for 5 days 5 h/day and infected with *P. aeruginosa*. FABP5^−/−^ mice reconstituted with Lenti-FABP5 show a significant decrease in lung tissue inflammation (Fig. 4b, c), inflammatory cells (Fig. 4d) and inflammatory cytokines (Fig. 4e) as compared to FABP5^−/−^ mice reconstituted with Lenti-Control. Taken together, these results suggest that FABP5 is critical to prevent CS and infection-induced pulmonary inflammation.

**Fig. 5:**
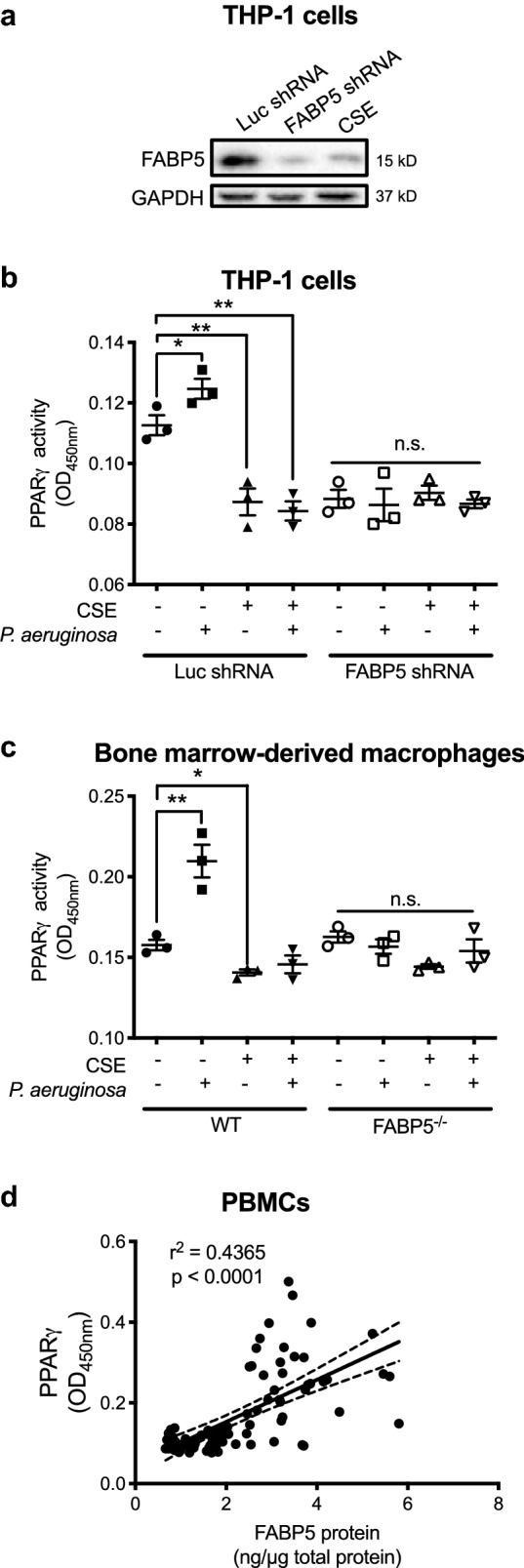
FABP5 expression is required for infection-induced PPARγ activation. **a** Western blot of FABP5 in THP-1 cells showing that cigarette smoke extract (CSE) reduces FABP5 expression to a similar extent as shRNA-mediated FABP5 knockdown. **b** PPARγ activity measured in shRNA-mediated FABP5 knockdown THP-1 cells treated with CSE and *P. aeruginosa* using an ELISA-based TransAM PPARγ activation assay. **c** PPARγ activity measured in bone marrow derived macrophages from WT or FABP5^−/−^ mice treated with CSE and *P. aeruginosa*. Data are representative of 3 independent experiments and represent the mean ± SEM. **d** FABP5 protein concentration and PPARγ activity correlation in PBMCs of non-COPD and COPD patients. *p < 0.05, **p < 0.01. *ns* non-significant

The studies described above demonstrate that CS exposure decreases FABP5 expression, which we have shown previously modulates PPARγ activity in airway epithelial cells [11]. Thus, we next hypothesized that reduced FABP5 expression leads to reduced PPARγ activity in macrophages and thus may lead to overt inflammation. We thus exposed THP-1 cells, a human monocytic cell line, to cigarette smoke extract (CSE) and used a lentiviral approach to knockdown FABP5. Interestingly, CSE resulted in FABP5 down regulation to a similar extend as FABP5 shRNA-mediated knockdown (Fig. 5a). We measured PPARγ binding to its consensus sequence in those same cells. While *P. aeruginosa* infection increased PPARγ activity, CSE greatly reduced PPARγ activity even in the presence of bacterial infection. PPARγ activity was reduced in cells treated with FABP5 shRNA-mediated knockdown (Fig. 5b). Similar PPARγ activity results were obtained in bone marrow-derived macrophages from WT and FABP5^−/−^ mice (Fig. 5c). Finally, we measured FABP5 protein levels and PPARγ activity in PBMCs of non-COPD and COPD patients and show a positive correlation between FABP5 protein levels and PPARγ activity (Fig. 5d). Taken together, these results suggest that FABP5 is required for induction of PPARγ activity, which in turn may regulate anti-inflammatory processes.

## Discussion

COPD exacerbations are triggered primarily by bacterial and viral infections and are associated with increased airway inflammation. This study provides new insights into the anti-inflammatory role of FABP5 in COPD exacerbations. Although FABP5 has been shown to be pro-inflammatory in several models, including high fat diet-induced skin inflammation [15], allergic asthma [16], LPS-induced liver damage [17] and neuropathic pain in mice [18], in this study we show that in the lungs of mice exposed to CS and infection, FABP5 expression, or re-expression, decreases inflammation. Ultimately, understanding how we may alter inflammatory responses in the lungs of CS-exposed mice will hopefully translate into promising pharmaceutical interventions that may be used in the treatment of bacterial-induced COPD exacerbations.

We have previously shown that FABP5 is decreased in COPD [10] and in airway epithelial cells exposed to CS [11, 19]. Here we report that FABP5 expression is decreased in PBMCs of patients with COPD and further decreased in patients reporting one or more episodes of COPD exacerbation. These findings confirm that COPD is not only an airway disease, but also a systemic disorder that involves systemic inflammatory manifestations [20, 21]. We also confirmed that CS decreases FABP5 mRNA and protein expression in WT mouse lung tissues.

COPD is a very complex disease that includes emphysema, bronchitis, and bronchiolitis. All aspects of the disease are impossible to recapitulate in one mouse model of COPD. A previous study has examined the concomitant effect of CS exposure and *P aeruginosa* infection, and showed that CS affects respiratory immune-inflammatory responses elicited by bacteria [22]. Using whole-body CS exposure in WT and FABP5^−/−^ mice to test the hypothesis that FABP5 is required for dampening CS- and infection-induced inflammation, we show that FABP5^−/−^ animals mount an exaggerated inflammatory response to *P. aeruginosa* infection following CS exposure. Furthermore, we were able to dampen the inflammatory response by re-expressing FABP5 in FABP5^−/−^ mice using a lentiviral approach.

Being at the interface between the host and the environment, the airway epithelium represents the first line of host defense against potential pathogens or irritants (such as cigarette smoke). This barrier not only provides the organism physiological function, but also protects it from damaging agents. The protective role of the epithelium includes recognition [23] and response to potentially dangerous particulates and microbes by producing various mediators such as proinflammatory cytokines (GM-CSF and IL-8), mucins (MUC5AC) and antimicrobial substances including peptides (β-defensins and LL-37) and proteins (lysozyme and lactoferrin) [24]. However, the persistent and repeated nature of infections within COPD patients indicates an abnormal epithelial cell function. We have previously shown that FABP5 is down regulated in primary airway epithelial cells of COPD patients as compared to patients without COPD [10]. Furthermore, primary human airway epithelial cells, which are knocked down for FABP5 and exposed to cigarette smoke, exhibit higher inflammatory cytokines secretion [11]. It is, thus, highly possible that the epithelial cells are also contributing to the increased inflammatory response seen in FABP5^−/−^ mice.

Interestingly, Perera et al. demonstrated that persistence of increased airway inflammation during exacerbation of COPD is associated with a prolonged symptom recovery time [25]. Thus, our FABP5^−/−^ mice exposed to CS and infected with *P. aeruginosa* may recapitulate an episode of exacerbation with heightened levels of inflammatory cells and cytokines. Further investigations are required to fully understand the implication of FABP5 in antimicrobial immunity during COPD exacerbations. In particular, studies looking at the effect of viral infection following the onset of bacterial infection are missing. Indeed, patients who develop recurrent exacerbations may be more susceptible to viral respiratory infections [26]. In addition, secondary bacterial infection is a common life-threatening event following influenza A virus infection. A thorough understanding of the events leading to susceptibility to this dual infection is lacking. We have shown that FABP5 plays a protective role in response to both influenza A virus (IAV) [27] and now *P. aeruginosa* infections, but the role of FABP5 in response to a dual IAV/*P. aeruginosa* infection has not been demonstrated, nor has the impact of CS been studied in this model.

Fatty acids, in addition to providing cell energy and structure, participate in a variety of cellular processes by modulating the activity of PPARs [28, 29]. PPARs are ligand-dependent transcription factors and members of the nuclear receptor superfamily [30]. Since fatty acids are hydrophobic, they rely on FABPs to exert their signaling effects [31]. FABP5 has been shown to stimulate PPARβ/δ transactivation in a breast cancer cell line (MCF-7 cells) [32]. Here, we investigated the functional cooperation between FABP5 and PPARγ in a monocytic-derived cell line and in human PBMCs in response to CS exposure and infection. PPARγ has been shown to have anti-inflammatory properties [33, 34], and as such has been targeted for therapeutic interventions in COPD [35]. In this study, we are showing an interesting association between FABP5 expression and PPARγ activity. Decreased FABP5 expression, either by CS exposure or by shRNA-mediated downregulation, lead to decreased PPARγ activity. We also show a positive correlation between FABP5 protein levels and PPARγ activity in human PBMCs. PPARγ has long been studied for its anti-inflammatory role, since PPARγ agonists were shown to dampen macrophage activation in vitro [36, 37]. Other studies have linked PPARγ with the development of pro-resolving macrophages [38] and resolution of inflammation [34]. It is, thus, tempting to speculate that FABP5 may exert its anti-inflammatory or pro-resolving properties through PPARγ activation. Further studies are required to confirm this hypothesis.

At this time, we do not know if this CS-induced FABP5 inhibition is reversible. Our human data would suggest that it is not, since COPD patients that quit smoking still have lower levels of FABP5. Our data also suggest that the level of FABP5 suppression may be an important factor of COPD exacerbations, and polymorphisms or other alterations that contribute to FABP5 decrease may contribute to disease susceptibility. Whether decreased FABP5 is a risk or a consequence of COPD exacerbation still remains to be elucidated.

## Conclusions

In summary, our study shows that FABP5 promotes the resolution of CS and infection-induced inflammation and that it may do so through PPARγ activation. Understanding the role of FABP5 during infections in the presence of CS may open up new therapies for the treatment and/or prediction of recurrent exacerbations of COPD.

## Methods

### Study population

Blood samples from the COPDGene cohort were used in this study. COPDGene (http://www.COPDGene.org) is a National Heart Lung Blood Institute-funded multicenter observational study designed to identify the genetic risk factors associated with COPD. The primary inclusion criteria are self-identified racial/ethnic category as either non-Hispanic white or African-American. Cases are diagnosed by post-bronchodilator spirometry as GOLD Stage II or greater (FEV1 < 80% predicted and FEV1/FVC < 0.7); smoking controls have normal spirometry. Both cases and controls are between ages 45 and 80 and have at least 10 pack-years of smoking history, and can be current or former smokers [14]. Peripheral blood mononuclear cells (PBMCs) from 20 non-smoker controls, 20 smoker controls and 48 patients with COPD were freshly isolated using CPT tubes (BD Biosciences, Vacutainer CPT tubes). Briefly, PBMCs were separated by density gradient centrifugation, using CPT tubes. After washing in HBSS, PBMCs were lysed to perform either *FABP5* quantitative real time PCR, FABP5 protein ELISA or PPARγ activity assay.

### THP-1 cells

The human monocytic THP-1 cells were obtained from ATCC (TIB-202) and were cultured in RPMI-1640 supplemented with 10% heat-inactivated fetal bovine serum (FBS), beta mercaptoethanol (0.05 mM), penicillin (100 U/ml), and streptomycin (0.1 mg/ml). Monocyte-derived macrophages were obtained by differentiation with PMA (100 nM) for 48 h.

### Cigarette smoke extract (CSE)

Two filtered research grade tobacco cigarettes (3R4F) from the Kentucky Tobacco Research and Development Center (University of Kentucky, Lexington, KY) were smoked through an apparatus with a constant airflow (0.2 l/min) driven by an air compressor. The smoke was bubbled through 20 ml of PBS and the pH was adjusted to 7.4. The CSE obtained was filtered and considered 100% CSE. It was further diluted to treat the cells with 2%. Cell viability was affected by CSE concentrations above 2%. The CSE was prepared freshly before each experiment along with an air control extract (AC) consisting of ambient air bubbled into 20 ml of PBS.

### RNA extraction and real time RT-PCR

RNA was extracted (QIAGEN), 10 ng/µl RNA was used with the Taqman RNA-to-Ct 1-Step kit (Applied Biosystems) to perform Real Time RT-PCR as previously described on GAPDH (Hs03929097_g1, amplicon length 58) and FABP5 (Hs02339439_g1, amplicon length 91) [39]. GAPDH was used as the housekeeping gene. The threshold cycle was recorded for each sample to reflect the mRNA expression levels. The comparative threshold cycle method was used to demonstrate the relative expression level of the gene of interest.

### FABP5 protein quantification

FABP5 protein concentrations were measured by ELISA in triplicates for each sample (antibodies-online Inc). Total extracted protein in the cell lysate were measured using the bicinchooninic acid (BCA) assay (Pierce). Results from the ELISA are expressed in ng FABP5/µg extracted protein out of 200 µl of cell lysate.

### PPARγ activity assay

PBMCs, THP-1 cells or bone marrow-derived macrophages were homogenized in nuclear protein extraction buffer to extract nuclear proteins following manufacturer’s instructions (Active Motif, Carlsbad, CA). Nuclear proteins (20 µg per sample) were used to perform PPARγ ELISA (Active Motif, Carlsbad, CA) to quantify PPARγ activation. Results are expressed as absorbance read at 450 nm with a reference wavelength at 650 nm.

### Animals

FABP5^−/−^ mice and littermate wild type (WT) controls, on a C57BL/6 J background, were kindly provided by Dr. Gokhan Hotamisligil at Harvard University (Boston, MA), and bred in our Biological Resources Center. Mice were kept on a 12-h light–dark cycle with *ab libido* access to food and water. All experimental animals used in this study were covered under protocols approved by the Institutional Animal Care and Use Committee of National Jewish Health.

### Cigarette smoke exposure protocol

8- to 12-weeks old WT and FABP5^−/−^ mice were exposed to room air or to the smoke from non-filtered research cigarettes (2R4; University of Kentucky, Lexington, Kentucky, USA), 5 h a day for 5 consecutive days using Teague-10 smoke chamber. The mice were exposed to a mixture of mainstream (11%) and sidestream (89%) cigarette smoke with a carbon monoxide (CO) concentration of 190 to 300 ppm and a total suspended particle (TSP) of 85 to 120 mg/m^3^ [40]. Between exposures, mice were housed in a holding room with circulating filtered air and given free access to water and standard rodent chow. Control mice were exposed to filtered air in separate TE-10 chambers.

### *P. aeruginosa* infection

One hour after the fifth day of room air or CS exposure, mice were anesthetized by ketamine (80 mg/kg body weight) and xylazine (10 mg/kg body weight), and then inoculated intranasally with 50 µl of 1 × 10^7^ colony forming units (CFU) of *P. aeruginosa* (PA01). As a control, mice were inoculated with 50 µl of saline. Sixteen hours post-infection, mice were sacrificed to examine bronchoalveolar lavage (BAL) cell profiles, lung tissue histopathology, and FABP5 expression. Five to six mice were harvested per group in three independent experiments.

### BAL and lung tissue processing

Mice were euthanized by intraperitoneal injection of Fatal Plus 2 µl/g of body weight and tracheotomized. The lungs were lavaged once with 1 ml phosphate-buffered saline (PBS). Cell-free BAL fluid was stored at − 80 °C for cytokine measurements. BAL cell cytospins were stained with the Diff-Quick Stain Kit (IMEB, Inc., San Marcos, California) for cell differential counts. Lung lobes were used for histology, RNA extraction and Western blot.

### Lung histopathology

Lungs were fixed in 10% phosphate-buffered formalin, dehydrated, embedded in paraffin, and cut at 4 μm thickness. Hematoxylin and eosin-stained lung sections were evaluated in a double-blinded fashion under the light microscope using a modified histopathologic inflammatory scoring system as described previously [41]. A final score per mouse on a scale of 0–10 (least to most severe) was obtained based on an assessment of the quantity and quality of peribronchiolar and peribronchial inflammatory infiltrates, luminal exudates and perivascular infiltrates.

### Cytokine measurements

Cytokine concentrations in mouse bronchoalveolar lavage fluid (BALF) samples were measured using the MSD Mouse Th1/Th2 9-plex Ultrasensitive kit (Meso Scale Discovery, Gaithersburg, MD) per manufacturer’s instructions.

### In vivo FABP5 overexpression

To overexpress FABP5 in vivo in alveolar macrophages, we integrated mouse FABP5 cDNA (Origene, MD, USA) into a lentiviral vector backbone (System Biosciences Inc., CA, USA). System Biosciences Inc. offers an HIV-based lentiviral expression system that consists of 3 main components: To package the lentiviral vectors we used the Lentivector packaging kit and the virus precipitation solution according to the manufacturer’s instructions. The lentiviral titer kit was used to determine the amount of viral vectors according to the manufacturer’s instructions. Intranasal administration of either the lenti-FABP5 or lenti-control vectors was performed on 6-weeks old FABP5^−/−^ mice. The amount of 1x10^8^ TU of lenti-FABP5 or lenti-control vectors were administered per mouse.

One week after intranasal treatment, animals were exposed to CS. On the last day of air or CS exposure, animals (5 mice/group) under anesthesia were intranasally infected with *P. aeruginosa* (10^6^ CFU/mouse) or saline (control) and sacrificed 16 h later. Tissues were evaluated for inflammation or processed for histology as described previously.

### Immunofluorescence

We used immunofluorescence to quantify FABP5 re-expression in lung tissue from FABP5^−/−^ mice treated with Lenti-FABP5. Immunofluorescence was performed on lung tissue sections fixed in 4% paraformaldehyde, embedded in paraffin and sections cut at 4 μm. These sections were deparaffinized and rehydrated. Antigen retrieval was carried out and consisted of boiling slides in a microwave pressure cooker (Tender Cooker; NordicWare) for 10 min in 0.01 M citrate buffer (pH 6.0). After blocking with 10% normal horse serum (Jackson ImmunoResearch; West Grove, PA) in PBS for 1 h, tissue sections were incubated with goat anti-mouse FABP5 (R&D) and rat anti-mouse CD68 (Biolegend) antibodies. The secondary antibodies Alexa Fluor 488 anti-goat IgG and Alexa Fluor 647 anti-rat IgG (Invitrogen) were applied for 1 h. Sections were mounted with Vectashield medium containing DAPI and cells were analyzed using a Zeiss LSM 700 confocal microscope and Zen Black software package (Carl Zeiss MicroImaging) with 10 × 63 magnification. The lentivirus efficiency of 42% was determined by the ratio of macrophages expressing FABP5 (number of positively double stained cells) per total number of macrophages (red cells).

### Bone marrow-derived macrophages (BMDM)

BMDM were generated in vitro by flushing bone marrow from mouse tibias and femurs. Progenitor cell suspensions were cultured for 5 days in DMEM containing 10% FBS, 100 U/ml penicillin, 100 µg/ml streptomycin and 10% L929 cell-conditioned medium as a source of CSF-1. Macrophage differentiation was confirmed by flow cytometry (> 95% positive for F4/80 and CD11b).

Human macrophages were transduced with either pLL3.7-shFABP5 or pLL3.7-shFirefly luciferase as previously described [11].

### Statistics

Data are expressed as mean ± SEM. One-way analysis of variance was used for multiple comparisons, and Tukey’s post hoc test was applied where appropriate. Student’s *t* test was used when only two groups were compared. Differences were considered statistically significant when *p* < 0.05.

## Abbreviations

BAL: bronchoalveolar lavage
*C. elegans*: *Caenorhabditis elegans*
COPD: chronic obstructive pulmonary disease
CS: cigarette smoke
ELISA: enzyme-linked immunosorbent assay
FABP5: fatty acid binding protein 5
FEV1: forced expiratory volume in 1 s
FVC: forced vital capacity
IAV: influenza A virus
IL-6: interleukin 6
KC: keratinocyte chemoattractant
mRNA: messenger RNA
PBMCs: peripheral blood mononuclear cells
PPARγ: peroxisome proliferator-activated receptor γ
*P. aeruginosa*: *Pseudomonas aeruginosa*
RNAi: RNA interference
RT-PCR: reverse transcription polymerase chain reaction
WT: wildtype
